# Effects of low level laser on periodontal tissue remodeling in hPDLCs under tensile stress

**DOI:** 10.1007/s10103-023-03885-0

**Published:** 2023-10-11

**Authors:** Jiaxi Zhang, Xizhong Zhang, Kaifang Han, Xuan Wang, Ziyuan Guo, Qi Deng, Jiahui Li, Shuxin Lv, Wenwen Yu

**Affiliations:** 1 Department of Orthodontics, Tianjin Stomatological Hospital, School of Medicine, Nankai University, Tianjin, 300041 China; 2 Tianjin Key Laboratory of Oral and Maxillofacial Function Reconstruction, Tianjin, 300041 China; 3 Department of Stomatology, Tianjin Beichen Hospital, Tianjin, 300400 China

**Keywords:** Periodontal ligament cells, Tension stress, Laser, Tissue remolding, ROS, Ca^2+^

## Abstract

This study aimed to investigate the effect of Low-Level Laser Therapy (LLLT) on human Periodontal Ligament Cells (hPDLCs) under tension stress. Primary hPDLCs were obtained using the tissue culture method, and P3 cells were utilized for the subsequent experiments. The study comprised four groups: a blank control group (Group B), a laser irradiation group (Group L), a tension stress group (Group T), and a laser + tension stress group (Group LT). Mechanical loading was applied using an in-vitro cell stress loading device at a frequency of 0.5 Hz and deformation of 2% for two hours per day for two days. Laser irradiation at 808 nm GaAlAs laser was administered 1 h after force loading. Cell samples were collected after the experiment. Bone and fiber remodeling factors were analyzed using PCR and Western blot. Flow cytometry was employed to assess the cell cycle, while ROS and Ca^2+^ levels were measured using a multifunctional enzyme labeling instrument. The results revealed that laser intervention under tension stress inhibited the expression of osteogenic differentiation factors, promoted the expression of osteoclast differentiation factors, and significantly increased the production of collagen factors, MMPs, and TIMPs. The LT group exhibited the most active cell cycle (P < 0.05). LLLT not only enhanced Ca^2+^ expression in hPDLCs under tension stress, but also stimulated the production of ROS. Overall, our findings demonstrate that LLLT effectively accelerated the proliferation of hPDLCs and the remodeling of periodontal tissue, possibly through the regulation of ROS and Ca^2+^ levels in hPDLCs.

**Supplementary Information:**

The online version contains supplementary material available at 10.1007/s10103-023-03885-0.

## Introduction

In orthodontic treatment, the acceleration of tooth movement is often desired to reduce the treatment duration. However, after the completion of orthodontic treatment, it is crucial to stabilize the tooth position and prevent tooth relapse. This process heavily relies on effective regulation of fiber and bone remodeling in the periodontal tissue. Enhancing periodontal remodeling has long been a challenging aspect of orthodontic treatment.

Previous research has primarily focused on pharmacological approaches to modulate periodontal tissue remodeling. Chemically modified tetracycline-3 (CMT-3) [1] has shown promise in stabilizing tooth position by improving the bone density in the mesial and distal alveolar bone. Raloxifene [2] has demonstrated the ability to inhibit osteoclast formation and promote bone volume. Aspirin [3] has been found to reduce the expression of TNF and IFN in the serum and periodontal ligament induced by orthodontic force. Orthodontic relapse can be mitigated by inhibiting the differentiation of CD^4+^ T cells and Th1 cells. Atorvastatin [4] has been shown to decrease tooth movement by upregulating OPG protein expression in the periodontal tissue and reducing the number of osteoclasts. While drug therapy offers advantages in terms of reduced invasiveness, it may also lead to serious systemic adverse reactions. Therefore, researchers have turned their attention to exploring physical methods. Low-level laser therapy (LLLT) has gained popularity in the field of dentistry due to its excellent penetration capability and safety profile [5].

Varella [6] conducted a study demonstrating that LLLT can accelerate the movement of canine teeth. Abdehameed [7] obtained similar results, showing that combining LLLT with micro-osteoperforations in canines could achieve the fastest movement speed. Kau [8] and Timothy [9] utilized pulsed LED devices in early-stage orthodontic treatment, resulting in an increased tooth movement speed of 1.12 mm/week compared to the control group's 0.49 mm/week. During the retention period following orthodontic treatment, researchers had reported that LLLT promotes the expression of MMP mRNAs, reduces the immunoreactivity of TIMP-1, thereby enhancing the immunoactivity of Col-I collagen, promoting the synthesis of periodontal fibers over degradation, and ultimately stabilizing teeth with fixed retainers [10]. Convisar [11] and Salehi [12] found that LLLT reduced tooth relapse by stimulating tissue remodeling and increasing bone density. In contrast to sagittal tooth movement, rotated tooth movements were more complex and prone to relapse. Clinical and animal experiments had shown that LLLT can significantly reduce the relapse of rotated teeth [13].

As a novel physical stimulation therapy for regulating tooth movement, the molecular mechanisms of LLLT are not yet fully understood. However, several hypotheses have been proposed. The mitochondrial theory [14] suggests that LLLT activates cytochrome c oxidase (CCO) at the end of the respiratory chain, leading to increased electron transfer and more ATP synthesis. The reactive oxygen species (ROS) theory [15] proposes that LLLT enhances oxidative potential, promotes ROS synthesis, and increases cellular redox activity, subsequently activating downstream pathways. According to the theory of calcium ions (Ca^2+^) theory [16], LLLT increases cell membrane permeability, resulting in an influx of Ca^2+^. Ca^2+^ can then transmit signals through CCO and nuclear factor-κB (NF-κB). As an important second messenger, Ca^2+^ participates in the mechanotransduction, influencing the cell's response to stimuli. Recent research has shown that Ca^2+^ is not only involved in mechanochemical signal transduction, but also plays a role in mediating the biological effects of LLLT, as blocking Ca^2+^ channels diminishes these effects [17].

Our previous studies demonstrated that LLLT could significantly reduce the retention period in rats and promote tooth stability [18]. Based on the aforementioned information, we hypothesize that LLLT can accelerate the speed of tooth movement during orthodontic treatment and reduce tooth relapse during the fixed retention period. To further investigate this, we examined the effects of LLLT on collagen and bone remodeling induced by hPDLCs under mechanical force and aimed to explore the possible underlying mechanisms.

## Materials and methods

### Cell culture

hPDLCs were isolated from the premolars extracted during orthodontic treatment from 12–17 years old teenagers. Informed consent was obtained from both the patients and their guardians, and the study was conducted under the guidance and approval of the ethics committee of Tianjin Stomatology Hospital. Tissue culture methods were employed to isolate hPDLCs, which were then cultured and passaged in 75cm^2^ culture flasks. The complete culture medium consisted of 10% FBS (Gibco, USA) and 1% penicillin–streptomycin (Gibco, USA). The cells were incubated in a humidified atmosphere with 5% CO_2_ at 37 °C. Passage 3 cells were used for subsequent experiments.

### hPDLCs Tensile stress model and LLLT irradiation

The experiment utilized a cell tensile stress loading device that was independently developed by Tianjin University of Technology. Once the cells adhered to the wall, tensile stress was applied at a frequency of 0.5 Hz and an elongation of 2% for a duration of 2 h per day, spanning a total of 2 days in both Group T and Group LT. An 808 nm GaAlAs diode laser with an output power of 48 mW, along with a fiber probe featuring a spot area of 0.785 cm^2^, was employed. The distance between the bottom of the silicone chamber and the laser source was maintained at 3 cm. An energy density of 2.45 J/cm^2^ was applied with an exposition time of 40 s. Laser irradiation was conducted in Group LT, one hour after the application of force loading, which followed the same protocol as Group L was in accordance with.

### Flow cytometry

For cell cycle detection, a cell cycle detection kit (Beyotime, China) was utilized following the manufacturer's instructions. Initially, hPDLCs were seeded in silicone chambers at a density of 3 × 10^6^ cells per chamber. To synchronize the cell cycle, the cells were pre-treated with a culture medium containing 2% FBS for 16 h. After the experimental treatment, the cells were collected and fixed overnight with 70% ethanol at -20 °C. The next day, the cells were washed twice with cold PBS and stained with a PI working solution (containing 100 μg/mL RNase A, 40 μg/mL PI, and 0.1% Triton X-100) at room temperature for 0.5 h in the dark. DNA content analysis was performed using a flow cytometer (Becton Dickinson, USA), and the data obtained was analyzed using the Flow Jo program.

### Real-time PCR

hPDLCs (5 × 10^6^/chamber) were seeded in a silicone chamber. After experimental treatment, the culture medium was removed, and the cells were collected for total RNA extraction using the SteadyPure Universal RNA Extraction Kit (Accurate Biology, China), according to the manufacturer's instructions. A cDNA reverse transcription was then conducted with Evo M-MLV RT Premix (Accurate Biology, China). Real-time PCR was performed with a Light Cycler/Light Cycler 480 Real-Time PCR System (Roche Diagnostics, Switzerland) using TB Green™ Premix Ex Taq™ I (Takara, Japan). Relative quantification was achieved by the comparative 2^−ΔΔCt^ method. Designed primer sequences were taken from the NCBI website. All primers were synthesized in Shengong, Shanghai. The sequences are listed in Table 1.

**Table 1:** Gene sequence

Gene	Sequence
GAPDH	F:5’-GCACCGTCAAGGCTGAGAAC-3’ R:5’-TGGTGAAGACGCCAGTGGA-3’
Runx2	F:5’-GAGATTTGTGGGCCGGAGTG-3’ R:5’-CCTAAATCACTGAGGCGGTC-3’
OCN	F:5’-CTCACACTCCTCGCCCTATTG-3’ R:5’-GCTTGGACACAAAGGCTGCAC-3’
NFATC1	F:5’-CCATGAAGTCAGCGGAGGAA-3’ R:5’-GAGGTCTGAAGGTTGTGGCA-3’
c-FOS	F:5’-GTCTCCAGTGCCAACTTCAT-3’ R:5’-CAGCCATCTTATTCCTTTCC-3’
CTSK	F:5’-CAGAATGGGAAGGTAGAGC-3’ R:5’-AACTGGAAGGAGGTCAGG-3’
MMP-1	F:5’-AGAGCAGATGTGGACCATGC-3’ R:5’-TTGTCCCGATGATCTCCCCT-3’
MMP-2	F:5’-ACCAGCTGGCCTAGTGATGAT-3’ R:5’-AAGGTGTTCAGGTATTGCATGT-3’
MMP-9	F:5’-CGCAGACATCGTCATCCAGT-3’ R:5’-AACCGAGTTGGAACCACGAC-3’
TIMP-1	F:5’-ATTCCGACCTCGTCATCAGG-3’ R:5’-GCATCCCCTAAGGCTTGGAA-3’
TIMP-2	F:5’-TAGTGATCAGGGCCAAAGCG-3’ R:5’-CTCAGGCCCTTTGAACATCTTT-3’
Col-I	F:5’-CCCTGAACTCTGCACCAAGT-3’ R:5’-GGGGTGGTAGAGTGGATGGA-3’
Col-III	F:5’-CCAGGAGCTAACGGTCTCAG-3’ R:5’-CAGGGTTTCCATCTCTTCCA-3’

### Weston blot assay and antibodies

The proteins of hPDLCs were harvested with a RIPA lysis buffer (Beyotime, China), and the protein concentration was measured using a BCA protein assay kit (Solarbio, China). Proteins were separated on SDS–PAGE (8–20% gel) and transferred to PVDF membranes. The membranes were blocked for 0.5 h with a blocking reagent, and then were washed and incubated with a primary antibody at 4 °C overnight. After several washes, the membranes were incubated with secondary antibodies (Beyotime, China) at room temperature for 1 h. ECL solution A and solution B (Beyotime, China) were mixed to image the membranes in a darkroom (Bio-Rad ChmiDoc, USA). The primary antibodies were listed in the following manner: GAPDH (ABclonal,1:5000,USA), NFATC1 (ABclonal,1:1000,USA), TIMP-1 (ABclonal,1:1000,USA),TIMP-2 (ABclonal,1:1000,USA), MMP-1 (ABclonal,1:1000,USA),MMP-2 (ABclonal,1:1000,USA), Runx2 (ABclonal,1:1000,USA),MMP-9 (ABclonal,1:1000,USA), COL-I (ABclonal,1:1000,USA), COL-III (ABclonal,1:1000,USA), c-Fos (CST, 1:1000, US), OCN (CST, 1:1000), CTSK (Abcam,1:1000).

### Intracellular ROS, Ca^2+^ using fluorescence microplate

hPDLCs (3 × 10^6^/well) were seeded in a silicone chambers. The cells were subjected to tensile stress for 1 h, followed by laser irradiation. Immediately after irradiation, the cells were harvested using trypsin. To detect ROS levels, the cells were exposed to DCFH-DA (1 μM) from the ROS detection kit (Beyotime, China). For Ca^2+^ release detection, Fluo-3/AM (1 μM) from Dojindo Laboratories was used. The cells were incubated at 37 °C for 20 min. After incubation, the cells were washed twice and resuspended in PBS. A total of 100 μl of the cell suspension was added to a fluorescence microplate. The fluorescence intensity was measured using a multifunctional enzyme labeling instrument (Tecan SPARK, Switzerland) with an excitation wavelength of 488 nm and an emission wavelength of 520 nm. Each group was evaluated in triplicate, and the levels of ROS and Ca^2+^ were represented by fluorescence intensity.

### Statistical analysis

Experiments were performed independently at least three times. The results were presented as the mean ± standard deviation and statistically analyzed with a one-way ANOVA, unless noted otherwise specified. A two-tailed P-value < 0.05 was considered statistically significant. Statistical analysis was conducted in SPSS version 20.0 (IBM).

## Results

### The effect of LLLT on the bone remodeling regulated by hPDLCs under tensile stress

**Fig. 1:**
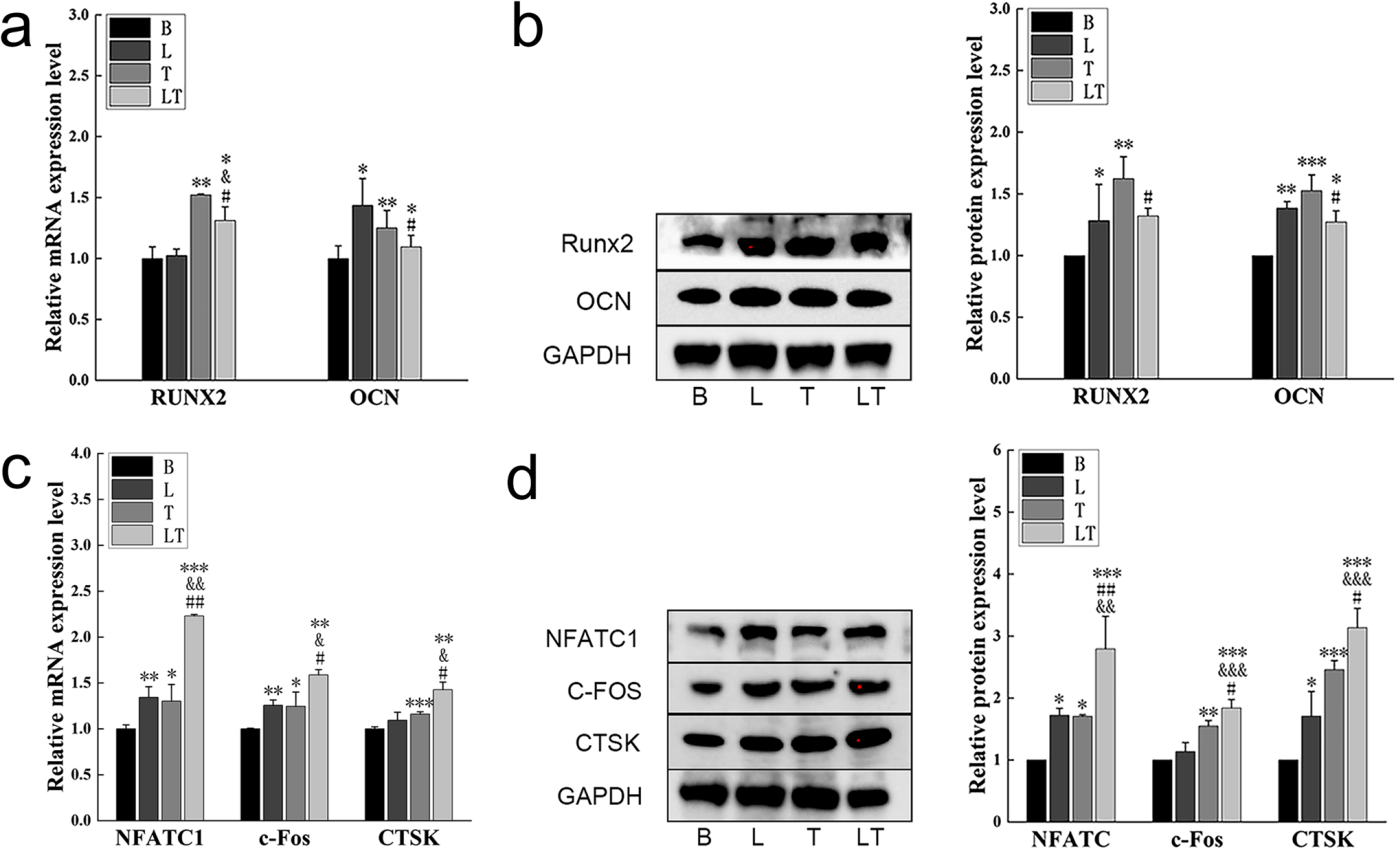
Osteogenic differentiated factors and Osteoclast differentiated factors detection (**a**) Quantitative real-time PCR analysis of Runx2 and OCN mRNA expression of hPDLCs. *n* = *3*. (**b**) Western blot analysis of Runx2 and OCN protein levels of hPDLCs. *n* = *3*. (**c**) Quantitative real-time PCR analysis of NFATC1, c-Fos and CTSK mRNA expression of hPDLCs. *n* = *3*. (**d**) Western blot analysis of NFATC1, C-FOS and CTSK protein levels of hPDLCs. *n* = *3*. Data represent the mean ± SD of three independent experiments. *P < 0.05 vs. the control group, #P < 0.05 vs. the tensile stress group and &P < 0.05 vs. the laser group by one-way ANOVA

To investigate the effect of LLLT on bone remodeling regulated by hPDLCs under tensile stress, real-time PCR and western blotting were performed to analyze the expression of osteogenic and osteoclastogenesis factors. The results demonstrated that LLLT had a significant impact on the bone remodeling. The mRNA and protein levels of osteogenic differentiation factors Runx2 and OCN were increased both in the laser group and tension stress group (except for Runx2 mRNA level in the laser group) (Fig. 1a, b). When laser irradiation was combined with tensile stress, the expression levels of Runx2 and OCN were also increased compared to the control group, but decreased compared to the tensile stress group.

Regarding osteoclast differentiation factors, the mRNA levels of NFATC1, c-Fos, and CTSK were increased in the laser group and tension stress group (Fig. 1c). A similar trend was observed in the protein levels of these osteoclastogenesis-related factors, except for CTSK mRNA level and c-Fos protein level in the laser group, where no significant difference was observed (Fig. 1d). The combination of LLLT and tensile stress showed a more pronounced effect. The expression levels of osteoclast differentiation factors were higher compared to each intervention applied separately.

### The effect of LLLT on collagen remodeling regulated by hPDLCs under tensile stress

**Fig. 2:**
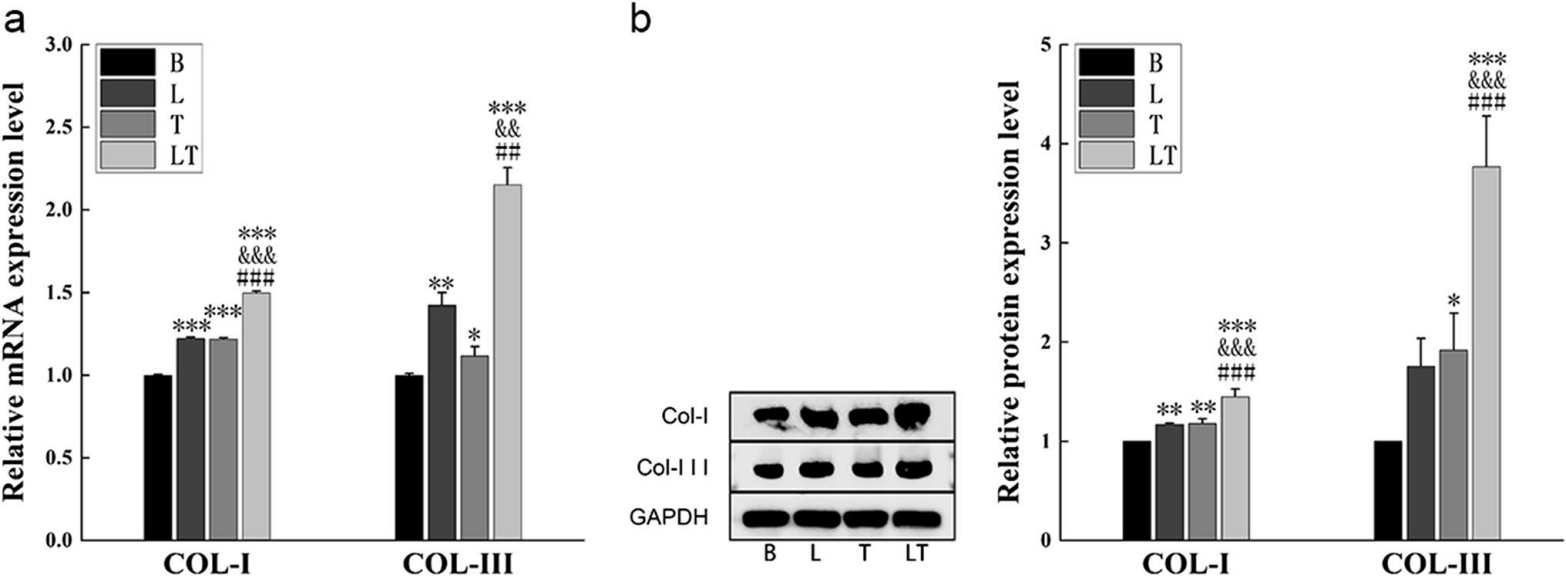
Collagen-modified factors detection (**a**) Quantitative real-time PCR analysis of Col-I and Col-III mRNA expression of hPDLCs. *n* = *3*. (**b**) Western blot analysis of Col-I and Col-III protein levels of hPDLCs. *n* = *3*. Data represent the mean ± SD of three independent experiments. *P < 0.05 vs. the control group, #P < 0.05 vs. the tensile stress group and &P < 0.05 vs. the laser group by one-way ANOVA

To investigate the effect of LLLT on collagen remodeling regulated by hPDLCs under tensile stress, this study examined the expression of main periodontal collagen fibers Col-I and Col-III, as well as matrix metalloproteinases (MMP-1, MMP-2, MMP-9) and their inhibitors TIMPs (TIMP-1, TIMP-2), which were involved in the regulation of collagen remodeling. The mRNA and protein levels of Col-I and Col-III significantly increased in both in the laser group and tensile stress group. When tensile stress was combined with laser irradiation, the mRNA and protein expression levels of Col-I and Col-III were further enhanced, surpassing those in the individual tensile stress group or laser group (Fig. 2).

**Fig. 3:**
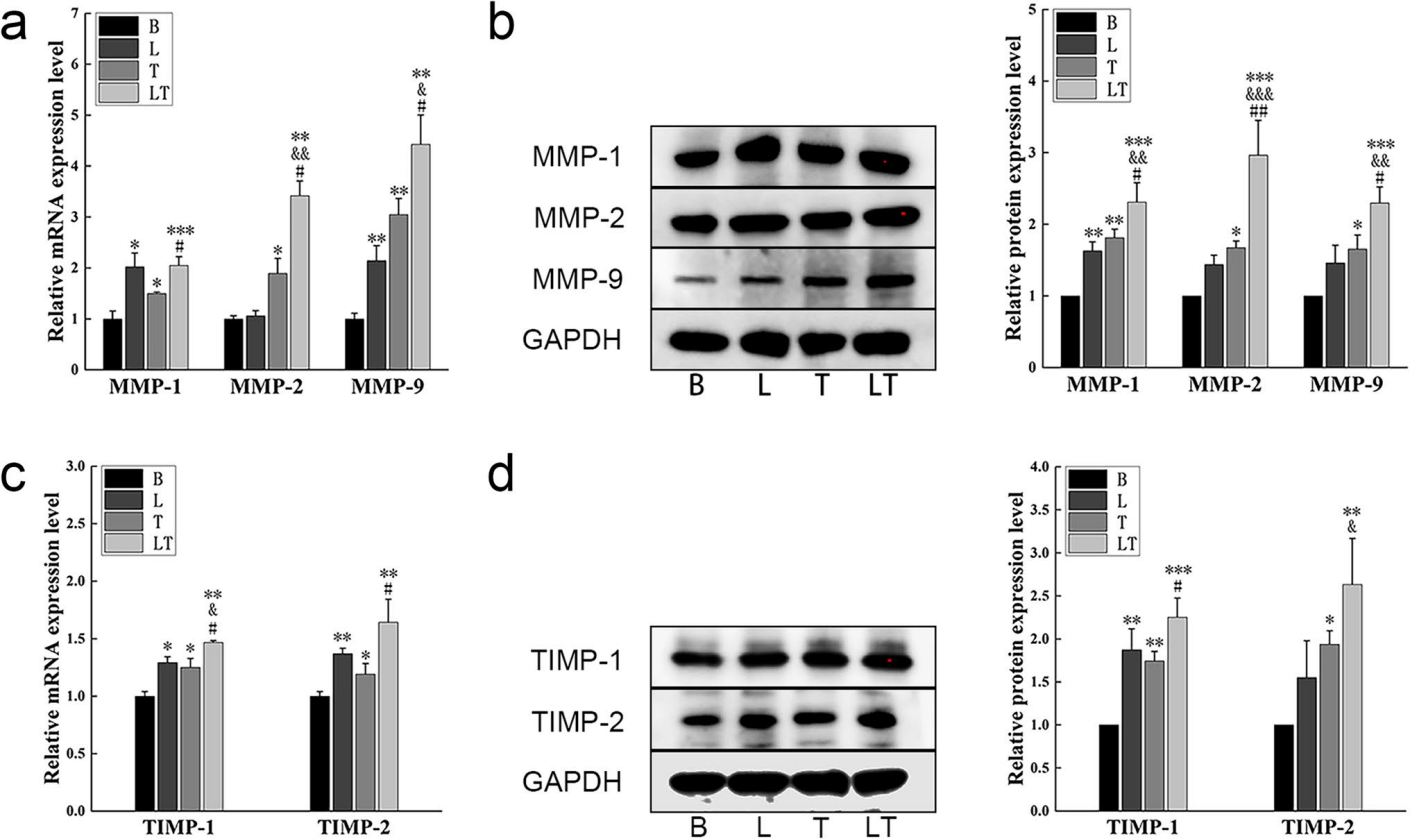
MMPs and TIMPs detection (**a**) Quantitative real-time PCR analysis of MMP-1, MMP-2 and MMP-9 mRNA expression of hPDLCs. *n* = *3*. (**b**) Western blot analysis of MMP-1, MMP-2 and MMP-9 protein levels of hPDLs cells. *n* = *3*.(**c**) Quantitative real-time PCR analysis of TIMP-1 and TIMP-2 mRNA expression of hPDLCs. *n* = *3*. (**d**) Western blot analysis of TIMP-1 and TIMP-2 protein levels of hPDLCs. Data represent the mean ± SD of three independent experiments. *P < 0.05 vs. the control group, #P < 0.05 vs. the tensile stress group and &P < 0.05 vs. the laser group by one-way ANOVA

The mRNA and protein expression levels of matrix metalloproteinases also showed a significant increase in the laser group and tensile stress group. However, the mRNA and protein expression of MMP-9 in the laser group did not exhibit consistent patterns. The combination of laser irradiation and tensile stress resulted in even higher promotion compared to each intervention applied alone (Fig. 3a, b). The expression of TIMPs followed a similar trend to that of MMPs, with an increase in the tensile stress group and a much higher level in the laser + tensile stress group. Similarly, the asynchrony between mRNA and proteins under laser irradiation was also observed in the expression of TIMPs. (Fig. 3c, d).

### The effect of LLLT on the cell cycle of hPDLCs under tensile stress

**Fig. 4:**
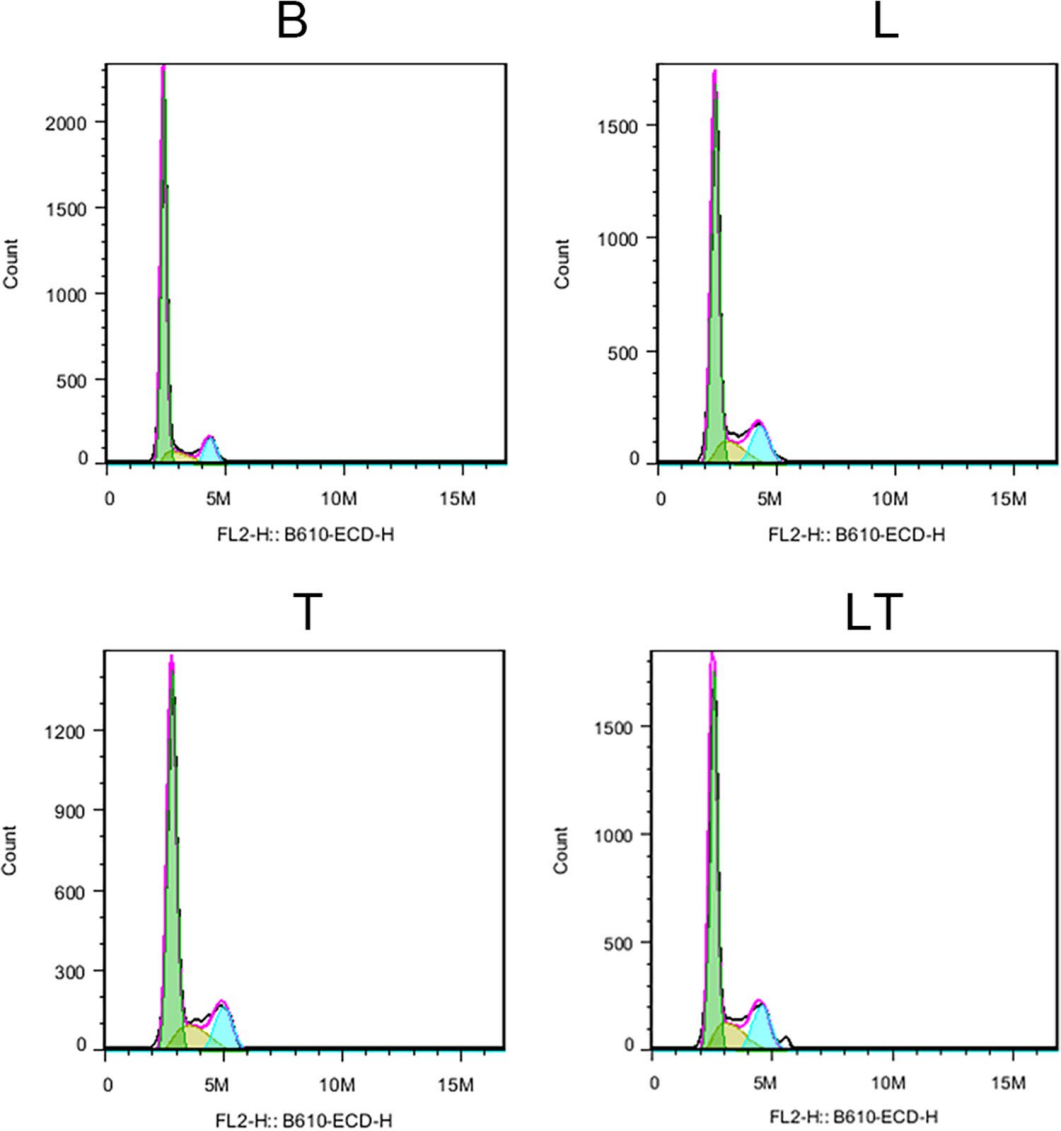
Cell cycle detection by FCM for 48 h. *n* = *3*

Under stationary growth conditions, a large proportion of the cells remained in the G0/G1 phase (74.91%) (Table 2 and Fig. 4). However, after LLLT and tension stress interventions, the number of cells in the G0/G1 phase decreased by 7.5% and 8.83%, respectively, while the number of cells in the S phase increased by 4.2% and 4.59%, respectively (Table 2). In comparison to the tension stress group and the laser group, the PI index of the laser + tension stress group increased, indicating that more cells passed through the G2/M checkpoint (Table 2 and Fig. 4). These results suggest that LLLT activates hPDLC proliferation under tension stress conditions.

**Table 2:** Cell cycle detection

	G1	S	G2	PI
Blank	74.91 ± 0.65	11.41 ± 0.27	11.34 ± 0.36	22.75 ± 0.63
laser	67.41 ± 0.26^***^	15.61 ± 0.83^***^	15.15 ± 0.5^***^	30.76 ± 1.33^***^
Tension stress	66.08 ± 0.06^***^	16.0 ± 0.57^***^	15.68 ± 0.7^***^	31.68 ± 1.27^***^
Laser + tension stress	61.75 ± 0.22^***###&&&^	18.11 ± 0.47^***##&&^	17.91 ± 0.17^***##&&&^	36.02 ± 0.64^***###&&&^

Cell cycle phase of each experimental group. *n* = 3. Data represent the mean ± SD of three independent experiments. *P < 0.05 vs. the control group, #*P* < 0.05 vs. the tensile stress group and &*P* < 0.05 vs. the LLLT group by one-way ANOVA

### The effect of LLLT on ROS and Ca^2+^ levels in hPDLCs under tensile stress

**Fig. 5:**
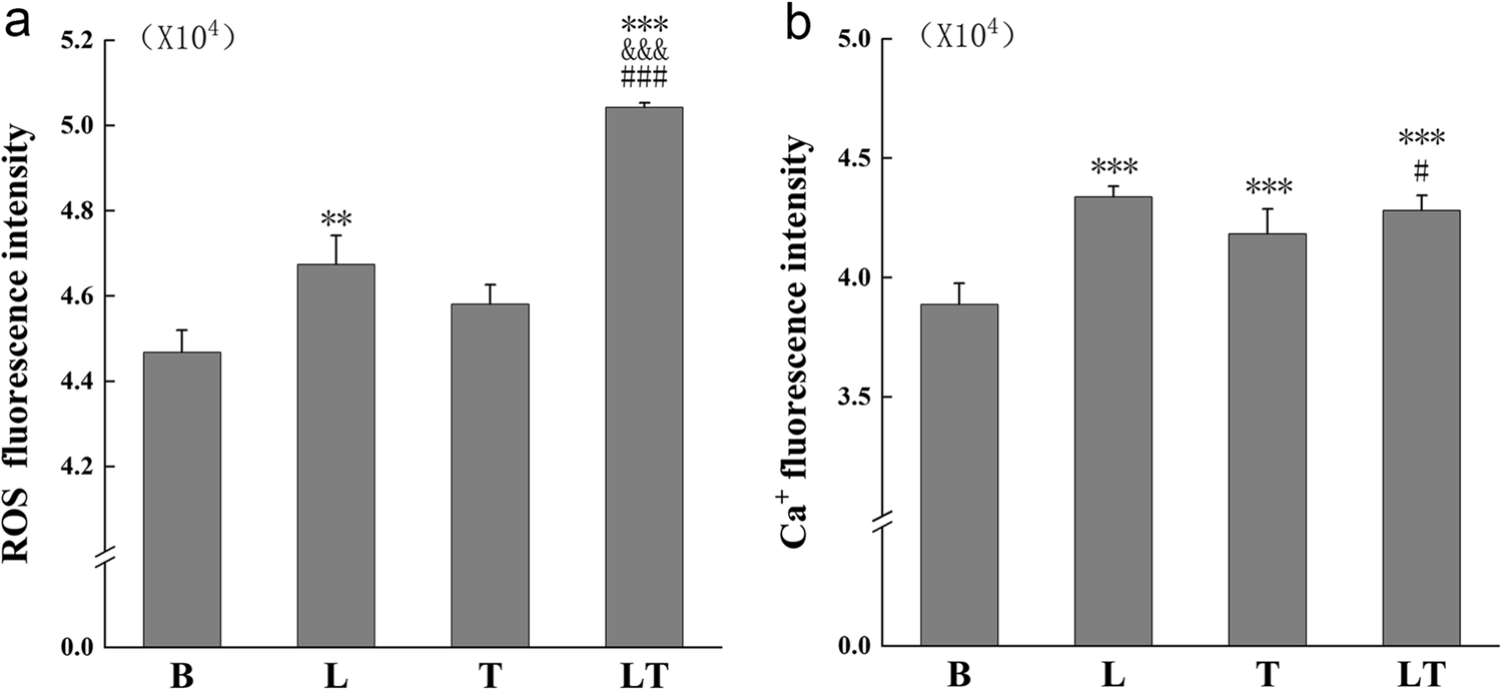
The level of ROS and Ca^2+^ detection (**a**) Determination of ROS content by multifunctional enzyme labeling instrument. *n* = *3*. (**b**) Determination of Ca^2+^ content by multifunctional enzyme labeling instrument. *n* = *3*. Data represent the mean ± SD of three independent experiments. *P < 0.05 vs. the control group, #P < 0.05 vs. the tensile stress group and &P < 0.05 vs. the laser group by one-way ANOVA

The multifunctional enzyme labeling instrument was used with ROS fluorescent probe and Fluo-3/AM was used to assess ROS generation and Ca^2+^ levels. The results revealed a significant increase in ROS levels in both in the laser group and the laser + tension stress group (P < 0.05), with the latter group exhibiting significantly higher ROS levels (P < 0.001). However, tensile stress alone did not appear to have a significant effect on ROS levels (P > 0.05) (Fig. 5a). In terms of Ca^2+^ level, all the experimental groups showed a noticeable increase compared to the control group, with the laser + tensile stress group exhibiting the highest Ca^2+^ level. (P < 0.05) (Fig. 5b).

## Discussion

Currently, LLLT is widely used in the field of orthodontics, but there is still a need for further research to understand its molecular mechanisms and determine the optimal application parameters. Among the different types of low-level lasers used in oral clinical practice, GaAlAs semiconductor lasers have several advantages, including small size, lightweight, ease of operation, and safe application. These lasers can directly affect deep tissues, promote microvascular expansion, enhance the oxygen-carrying capacity of red blood cells, stimulate various biological processes such as RNA and DNA synthesis, and promote protein secretion. As a result, they can enhance the cellular and molecular responses in tissue repair [19].

Orthodontic tooth movement (OTM) involves bone resorption on the compression side and new bone formation on the tension side in response to mechanical forces. This process relies on the proliferation and differentiation of various cells [20–22]. PDLCs play a crucial role as they are the primary sensors that respond to mechanical signals and regulate tissue remodeling [23]. Our results demonstrated that tension stress stimulation led to an increase in hPDLCs entering the DNA synthesis phase, providing a cell reserve for periodontal tissue remodeling. When combined with laser irradiation, more cells progressed through the cell division phase via the G2/M checkpoint, actively participating in periodontal remodeling [24, 25]. Accumulating evidence suggests that LLLT, when applied at appropriate doses, can promote cell proliferation and differentiation in various cell types, including bone marrow mesenchymal stem cells [26], stem cells from the apical papilla [27], and human adipose-derived stem cells [28].

The results obtained from PCR and Western blot analysis revealed that under tension stress, the expression of bone remodeling factors significantly increased, indicating the active involvement of hPDLCs in osteogenesis and osteoclast remodeling. However, the intervention of LLLT promoted the expression of osteoclast differentiation factors while inhibiting the expression of osteogenic differentiation factors. This observation suggests that there may be an asynchronous bone remodeling process occurring. Bone remodeling induced by mechanical force consists of three stages: bone resorption mediated by osteoclastogenesis, the release of cytokines during the process of bone resorption to induce osteoblast differentiation, and subsequent reversal of bone resorption was reversed, followed by osteoblast-mediated bone formation, ultimately completing the process of bone remodeling process. It is well known that bone resorption mediated by osteoclastogenesis is a rate-limiting step in tooth movement. Therefore, LLLT selectively stimulates the process of osteoclastogenesis, thereby accelerating the speed of the "bone resorption" stage. Previous studies have shown that LLLT significantly increases osteoclast activity during the initial stage of tooth movement, while having no significant effect on osteoblast activity [29–31].

It is important to note that the optimal parameters for LLLT can vary depending on the cell type being targeted. As mentioned earlier, the biphasic dose–response relationship of LLLT has been observed in in-vitro research and animal experiments. Moreover, the effective dosage range for different cell types is narrow and difficult to overlap [32]. Therefore, it is not surprising that the same irradiation parameters can lead to contrasting effects. Additionally, we observed an interesting phenomenon where the expression levels of certain factors did not show consistency between mRNA and protein levels. Karu has reported that the increase in reactive oxygen species (ROS) and cytosolic free calcium ion (Ca^2+^) concentration caused by LLLT can trigger conformational changes in proteins [33]. Furthermore, the biological effects of LLLT depend on the initial redox state of cells [32]. These factors may partially explain the inconsistent findings in our study, but further investigation is needed to elucidate the specific mechanisms involved.

MMPs (matrix metalloproteinases) play a crucial role in the remodeling of periodontal tissue by degrading the extracellular matrix (ECM) and regulating collagen fibers [34]. TIMPs (tissue inhibitors of metalloproteinases) are natural inhibitors of MMPs. The balance between MMPs and TIMPs determines the rate of bone matrix degradation [35]. In our experiment, under tension stress, the expression levels of MMP-1, MMP-2, and MMP-9 mRNA and protein in hPDLCs significantly increased, indicating active ECM remodeling. However, the expression of MMPs did not continue to increase indefinitely. The cells responded quickly with protective effects, leading to an increase in TIMPs expression to prevent excessive degradation of periodontal tissue. After laser intervention, significant differences were observed in protein levels, except for TIMP-2. The expression of both MMPs and TIMPs was more pronounced, suggesting that LLLT accelerated ECM remodeling.

The periodontal ligament consists of cells and extracellular matrix, with collagen fibers accounting for 70% of the protein components. Collagen I and III not only provide a structural framework for periodontal ligament reconstruction, but also participate in the regulation of cell proliferation, migration, and specific gene expression through specific amino acid sequences binding to integrin receptors on the cell surface under mechanical force [36–38]. The expression of Col-I and Col-III significantly increased under tension stress stimulation, and the intervention of LLLT enhanced the regulatory effect of mechanical force on collagen remodeling. Based on the previous experiments, we speculate that LLLT may have a greater promoting effect on TIMPs than MMPs, thereby reducing the degradation of collagen by MMPs and resulting in overall promotion of collagen. We also hypothesize that LLLT may have a greater impact on type III collagen, leading to a decrease in the collagen I/III ratio and an accelerated collagen remodeling process. However, it is important to note that the present study did not specifically examine the changes in the ratio of collagen I/III, and further detailed experiments are needed to clarify this hypothesis.

In summary, our study demonstrated that LLLT can promote the process of periodontal remodeling under tension stress. Previous studies have shown that the level of reactive oxygen species (ROS) determines its biological effects. ROS can act as a beneficial signal to accelerate cell metabolism at low levels, but as a harmful cytotoxic agent at high levels [39, 40]. Appropriate laser irradiation increases intracellular ROS levels and accelerates cell metabolism [41]. Based on our results, we speculate that the level of ROS developed by LLLT under tension stress is suitable for hPDLCs, as evidenced by the findings from the cell cycle, PCR, and Western blotting assays. However, we observed that tension stress had little effect on ROS levels, which may be attributed to the short duration of force application time and small deformation [42, 43].

Following mechanical stimulation, hPDLCs translate mechanical signals into chemical signals through mechanochemical signal transduction, thereby initiating subsequent cascade reactions. Previous research has indicated that Ca^2+^ is a second messenger in the process of mechanochemical signal transduction [44, 45]. Our experiments also confirmed that the tension stress altered the Ca^2+^ levels, with a more pronounced effect observed when laser irradiation was combined with tension stress. Changes in Ca^2+^ levels were detectable after one hour of force application. Our study suggested that the response of hPDLCs to LLLT was also involves the "Central Hub" of Ca^2+^. Recent research has discovered that the extracellular Ca^2+^ influx following laser irradiation may represent a novel pathway for activating cellular metabolism [17]. LLLT increases the level of Ca^2+^ in hPDLCs under tension stress, potentially accelerating mechanochemical signal transduction. Additionally, LLLT may exert photobiological stimulation through the increase of Ca^2+^. However, the precise mechanisms by which Ca^2+^ is involved and transferred from excitation to effect require further investigation.

## Conclusion

In this study, an in-vitro cell stress loading device was utilized to investigate the effect of LLLT on the regulation of periodontal tissue remodeling induced by hPDLCs under tension stress. The results provide evidence that LLLT has a positive impact on bone remodeling, collagen remodeling, and cell cycle in hPDLCs under tension stress. These findings contribute to our understanding of the potential mechanisms underlying the effects of LLLT and may inform its clinical application in orthodontic treatment. Further research is warranted to explore the detailed mechanisms of action of LLLT and to optimize its use in clinical practice.

### Supplementary Information

Below is the link to the electronic supplementary material.

Supplementary file1 (XLSX 11 KB)

## Data Availability

Data available on request.

## Declarations

### Informed content

Written informed consent was obtained from all patients.
